# Nitrogen-Containing Functional Groups-Facilitated Acetone Adsorption by ZIF-8-Derived Porous Carbon

**DOI:** 10.3390/ma11010159

**Published:** 2018-01-19

**Authors:** Liqing Li, Xiancheng Ma, Ruofei Chen, Chunhao Wang, Mingming Lu

**Affiliations:** 1 School of Energy Science and Engineering, Central South University, Changsha 410083, China; maxiancheng@csu.edu.cn (X.M.); ruofeichen@csu.edu.cn (R.C.); wangchunhao@csu.edu.cn (C.W.); 2 College of Engineering and Applied Science, University of Cincinnati, Cincinnati, OH 45221, USA; mingming.lu@uc.edu

**Keywords:** ZIF-8, porous carbon, nitrogen-containing functional groups, acetone adsorption

## Abstract

Nitrogen-doped porous carbon (ZC) is prepared by modification with ammonia for increasing the specific surface area and surface polarity after carbonization of zeolite imidazole framework-8 (ZIF-8). The structure and properties of these ZCs were characterized by Transmission electron microscopy, X-ray diffraction, N_2_ sorption, X-ray photoelectron spectroscopy and Fourier transform infrared spectroscopy. Through static adsorption tests of these carbons, the sample obtained at 600 °C was selected as an excellent adsorbent, which exhibited an excellent acetone capacity of 417.2 mg g^−1^ (25 °C) with a very large surface area and high-level nitrogen doping (13.55%). The microporosity, surface area and N-containing groups of the materials, pyrrolic-N, pyridinic-N, and oxidized-N groups in particular, were found to be the determining factors for acetone adsorption by means of molecular simulation with density functional theory. These findings indicate that N-doped microporous carbon materials are potential promising adsorbents for acetone.

## 1. Introduction

The emission of volatile organic compounds (VOCs) vapors has caused not only serious air pollution but also a large loss of valuable chemicals [1,2,3,4]. Therefore, there is growing concern about the development of effective adsorption technologies for VOCs. Adsorption is a promising and effective technology for removing VOCs [5,6]. Up to now, a wide variety of adsorbents have been explored for VOCs adsorption, including activated carbon [7,8], silica [9], carbon nanotubes [10] and metal-organic frameworks (MOFs) [11]. Among these adsorbents, porous carbon materials have enormous commercial potential due to their easy-to-control pore structures, larger surface areas, and good chemical/thermal stability in production and regeneration. 

Recently, MOFs have attracted extensive attention in application such as gas adsorption [12,13,14], sensing [15], environmental pollution treatment [16,17], etc. The MOFs have been used as a template and/or precursor to prepare porous carbon materials with novel structures and chemical properties, such as high surface area, large pore volume, tunable pore size and high content of heteroatoms [3,18,19], So far, a variety of MOFs, such as ZIF-8 [18,20], Al-PCP [21], and MOF-5 [19,22] have been proven to be promising self-sacrificial templates to produce nanoporous carbon materials. ZIF-8, which is readily prepared and rich in carbon and nitrogen, and can be used as an ideal precursor for preparation of nitrogen-doped porous carbon materials with high active site density and uniformly distributed catalytic centers [23]. 

N-doped carbon materials can be prepared by pyrolysis of N-containing precursors, or by heat treatment of carbon materials under NH_3_ atmosphere to introduce nitrogen into the carbon frameworks. The resulting N-doped carbons generally have excellent thermal stability and enhanced adsorption properties. Nitrogen functional groups increase the carbon surface polarity, basicity, and π electrons that have attracted extensive attention in application such as CO_2_ capture [24,25,26,27], electrochemical capacitance [28,29], lithium batteries [30,31] and ORR in fuel cells [32,33,34]. From the literature, sole N-doped carbon materials towards VOCs have not yet been reported.

In this work, ZIF-8 was used as precursor and template to prepare porous carbon, and NH_3_ modification was adopted to improve surface area and surface polarity of the carbon materials. Accordingly, resulting porous carbon introduces nitrogen functional groups from 2-Methylimidazole (HMeIM) anion in the ZIF-8 and NH_3_. The synergistic effects of nitrogen-containing surface functional groups of porous carbon on its acetone adsorption properties were investigated. 

## 2. Experimental Section

### 2.1. Material Synthesis

All chemicals were obtained commercially and used without further purification. 2-Methylimidazol (HMeIM, 98%) was purchased from Aladdin Chemicals, Shanghai, China. Ammonia solution (NH_4_OH, 33%), acetone (CH_3_COCH_3_, 99.5%), methanol (CH_3_OH, 98%) and zinc nitrate hexahydrate (Zn(NO_3_)_2_·6H_2_O 98%) were purchased from Xilong Chemicals Co., Ltd., Shantou, China. Activated carbon (AC) was purchased from ChangGe Carbon Industry Chemical Co., Ltd., Xuchang, Henan, China. N_2_ gas of ultra high purity (99.999%) and NH_3_ gas of high purity (99%) were purchased from High-tech Gas Ltd., Changsha, China.

ZIF-8 was prepared by stirring the mixture of HMeIM (4 g) and Zn(NO_3_)_2_·6H_2_O (7.2 g) in ammonia (180 mL) and methanol (72 mL) solutions for 5 h. Finally, the white powders were obtained by washing with methanol (30 mL) for three times, and drying under vacuum at 80 °C for overnight [35]. 

The synthesized ZIF-8 was evacuated at 250 °C for 2 h to get degassed ZIF-8 samples. Then the ZIF-8 was transferred into a quartz boat and placed in a furnace for carbonization. Subsequently, the furnace was heated to 900 °C for 2 h with 5 °C min^−1^ under N_2_ flow. After carbonization, the samples were washed with HCl solution (5%) vigorously and subsequently several times with distilled water, and dried under vacuum at 120 °C for overnight, denoted as ZC. Then, the ZC was treated through ammonia at 200, 400, and 600 °C for 2 h, affording the samples designed as ZCN200, ZCN400, ZCN600, respectively. The ZC and ammonia-treated ZC samples were labeled as ZCs.

### 2.2. Physicochemica-Characterization of the Samples

TEM images were recorded on a transmission electron microscope (Tecnai G2 20S-Twin, FEI Co., Hillsboro, OR, USA) with an operating voltage at 200 kV. X-ray diffraction (XRD) patterns were performed on a PANalytical powder diffractometer (Rigaku Corporation, Kyoto, Japan) at 40 mA and 40 kV for Cu Kα radiation. N_2_ adsorption isotherm was measured by using automatic volumetric adsorption equipment (SA3100, Beckman-Coulter Instrument Co., Los Angeles, CA, USA) at 77 K. The pore size distributions were obtained via a nonlocal density function theory (NLDFT) method using nitrogen adsorption data.The sample was ground to a powder, degassed at 100 °C under vacuum for 5 h, and then tested. Fourier transform infrared spectroscopy (FTIR) spectra was measured with a PerkinElmer-2000 FTIR spectrometer (Perkin Elmer, Foster, CA, USA) ranging from 4000 to 400 cm^−1^. X-ray photoelectron spectroscopy (XPS) analyses were carried out on a Shimadzu ESCA-3400 X-ray photoelectron spectrometer (Rigaku Corporation, Kyoto, Japan). The XPS measurements were performed using Mg Kα (1253.6 eV) radiation at 12 mA and 10 kV with a takeoff angle of 90°.

### 2.3. Adsorption Measurement

The isotherm adsorption of acetone was measured using a JW-BK132Z (Beijing JWGB Sci & Tech Co., Ltd., Beijing, China) automatic volumetric adsorption equipment. Prior to the adsorption measurement, the sample (around 50 mg) was evacuated at 150 °C for several hours. The acetone adsorption experiments were carried out at 25 °C [36].

### 2.4. Computational Details

All of the first principle calculations were conducted on the basis of density functional theory (DFT) with the use of the DMol^3^ code [37]. The energy of the functional group and the interaction of carbon dioxide was calculated using the DFT calculation coupled with the van der Waals correct correction (DFT-D) [38]. Perdew, Burke, and Ernzerhof (PBE) within the generalized gradient approximation (GGA-PBE) was selected [39]. The atomic orbit is described using double numeric polarization (DNP) basis set, which is comparable to 6-31G (d, p). The type of core processing is set up using a DFT half-core Pseudopots (DSPP) specifically designed for DMol^3^ calculations [40]. The real-space orbital global cutoff radius is 3.7 Å. The convergence threshold parameters for the optimization are 10^−5^ Hartree (energy), 2 × 10^−3^ Hartree (gradient), and 5 × 10^−3^ Hartree (displacement), respectively [41]. Therefore, BSSE effects need not be taken into consideration for calculating the energies. The acetone adsorption energy was calculated in the following manner:

$$E_{\mathrm{ads}}=E_{\mathrm{surface}+{\mathrm{CO}}_{2}}-\left(E_{\mathrm{surface}}+E_{{\mathrm{CO}}_{2}}\right)$$

where *E*_ads_, *E*_surface+acetone_, and *E*_acetone_ are adsorption energy and total energy of adsorption-adsobate complex, carbon surface, and isolated acetone, respectively. The binding energy of acetone in group-functionalized surface is a significant parameter for the acetone adsorption on the group-functionalized surface. An enhancement in the *E*_ads_ would be highly beneficial to the ZCs samples for capturing acetone.

## 3. Results and Discussion 

### 3.1. Textural and Chemical Properties of ZCs and AC

**Figure 1:**
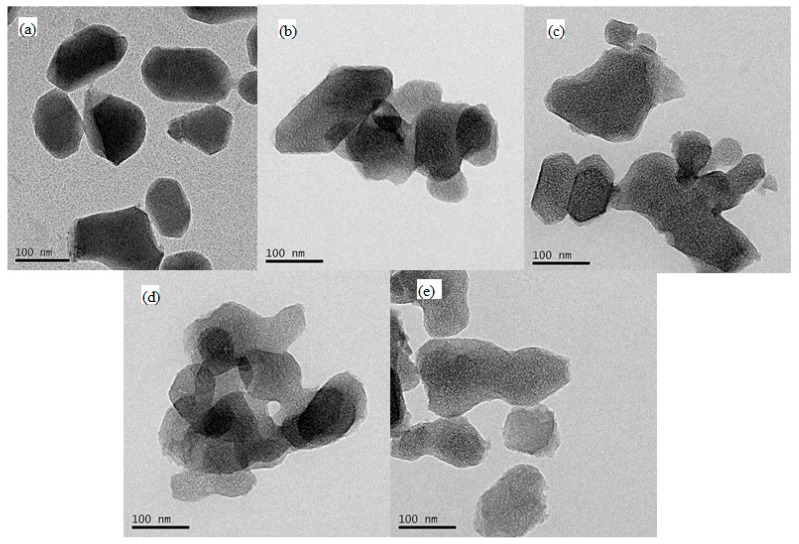
TEM images of the nitrogen doped and porous carbon (ZC) samples: (**a**) ZIF-8; (**b**) ZC; (**c**) ZCN200; (**d**) ZCN400 and (**e**) ZCN600.

The TEM images of the ZCs samples are shown in Figure 1. As shown in Figure 1b, the resulting porous carbons maintain a surface morphology similar to that of the parent ZIF-8 (see Figure 1a), revealing that the carbonized template do not change the surface morphology [42]. The slight differences observed in the surface morphology of ammonia treated with ZCs may be possibly attributed to reaction of NH_3_ with the carbon species, and the ammonia-treated ZC samples show different morphologies depending on the temperature.

**Figure 2:**
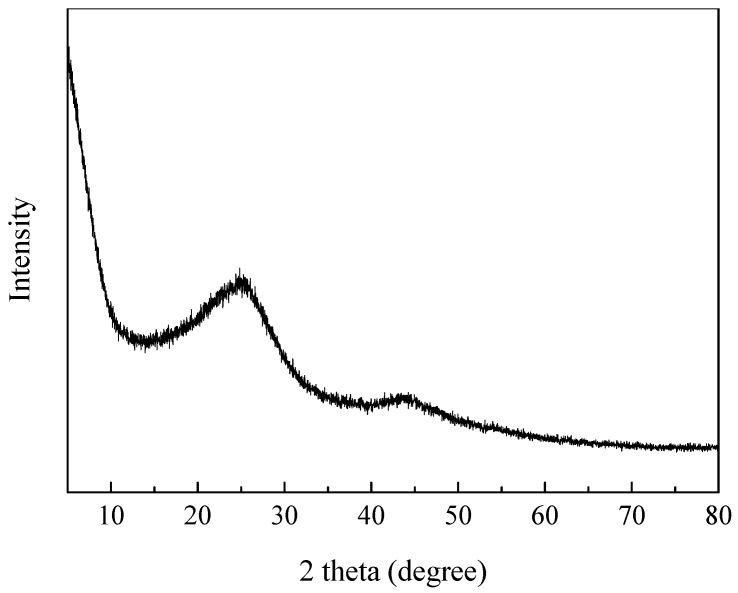
XRD profile of ZC sample.

Figure 2 displays the XRD pattern of ZC. Only two broad peaks of ZC are 25° and 44° corresponding to (100) and (101) diffractions peak of graphitic carbon, indicating the presence of few-layered graphene in the carbon materials [18]. In addition, the diffraction peak of the impurities cannot be observed, indicating that Zn on the surface of the carbon material is removed. During the carbonization process, Zn ions were reduced to Zn metals by carbon and then vaporized at 900 °C, and the other residual was removed by 5% HCl.

**Figure 3:**
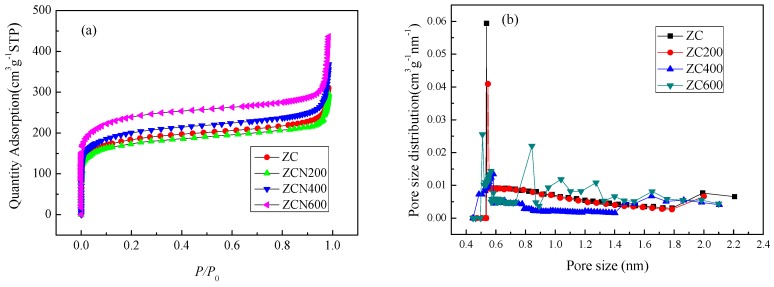
N_2_ adsorption isotherms at 77 K (**a**) and pore size distribution (**b**).

Figure 3 shows nitrogen adsorption isotherms of the ZCs samples. The ZC and the modified ZCs samples exhibit type IV isotherm profiles according to the (IUPAC) classification. For the ZC and the modified ZCs samples, the nitrogen adsorption sharp increase at low relative pressure and at high relative pressure indicates the presence of microporosity and macroporosity, respectively [43]. The pore size and the surface area of different materials are listed in Table 1. After carbonization of ZIF-8 at 900 °C, the specific surface area and total pore volume are 624 m^2^ g^−1^ and 0.46 mL g^−1^, respectively. After ZC ammonia treatment, at 200 °C and 400 °C, the specific surface area and the total pore volume did not change, whereas the highest values are after treated at 600 °C (805 m^2^ g^−1^ and 0.62 mL g^−1^). A significantly increase in microporosity was detected in the sample obtained by NH_3_ treatment at 600 °C, maybe attributing to ammonia decomposing to free radicals such as NH_2_, NH, and H at 600 °C. The gasification of carbon occurs in the form of methane, hydrogen cyanide and cyanogens due to the attacking from these radicals [44]. The pore size distribution of ZC samples is shown in Figure 3b. The pore size of the sample is mainly distributed around 0.55 nm. Compared with ZC, the pore size distribution of ZCN200 does not change. However, at 600 °C, this increase of larger microporous size may be attributed to ammonia decomposing to free radicals such as NH_2_, NH, and H at 600 °C.

**Figure 4:**
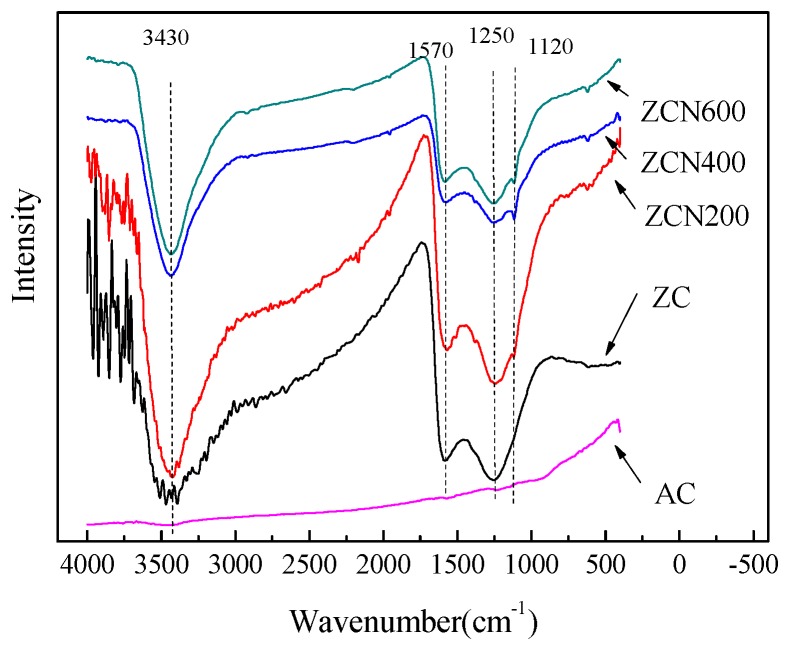
FTIR spectra of ZCs and AC.

The FTIR spectra of the ZC and the treated ZCs samples are presented in Figure 4. The ZC contains several peaks, the 1250 cm^−1^ assigned to C-N (at 1250 cm^−1^) [45], and 1570 cm^−1^ attributed to C=N and N–H groups [46,47]. At 200 °C ammonia treatment, the spectrum do no change. However, at 400 °C and 600 °C ammonia treatment caused some changes. From the spectral comparison of the ZCs samples, it was found that there was a difference at 1120 cm^−1^ due to the presence of C–O [48]. After heating to 400 °C under the ammonia atmosphere, it is observed that the C–O peak significantly increased. However, the amount of C–O groups decreased at 600 °C [49]. The weak bands at ca. 3430 cm^−1^ can be attributed to the presence of the N–H and O–H symmetric stretching vibration [44].

**Figure 5:**
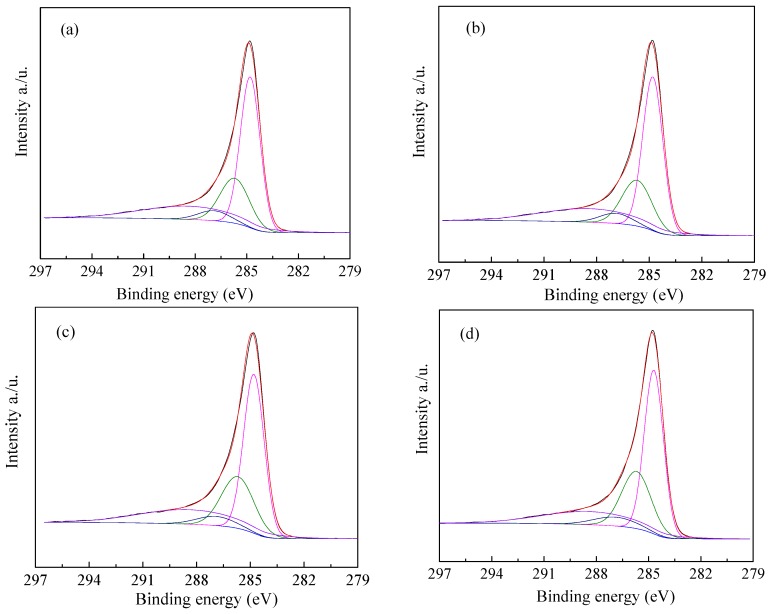
C1s spectrum of the ZC samples: (**a**) ZC; (**b**) ZCN200; (**c**) ZCN400; (**d**) ZCN600.

**Figure 6:**
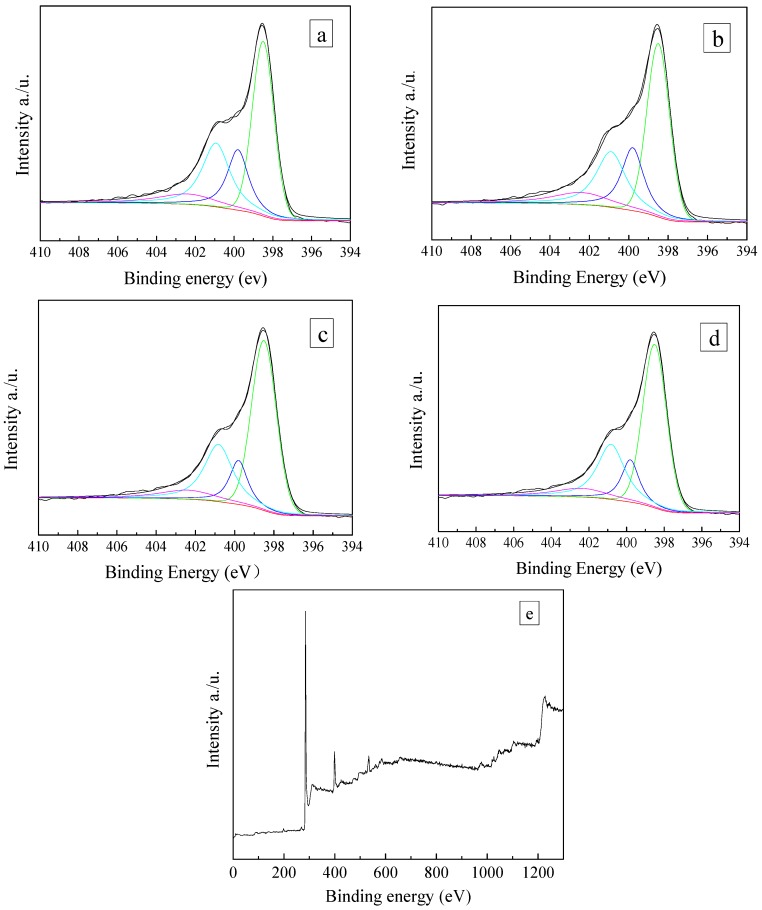
N1s spectrum of the ZC samples: (**a**) ZC; (**b**) ZCN200; (**c**) ZCN400; (**d**) ZCN600 and (**e**) XPS survey spectra of ZC.

The surface chemical properties of ZCs samples were analyzed by XPS. Figure 5 shows the C1s spectra. The peaks at 284.7, 285.7, 286.9 and 288.4 eV are attributed to C–C, C–N, C–O and C=N, respectively. The N1s spectra are shown in Figure 6. The deconvolutions of N1s photoelectron spectrum for samples and fitted by four separate peaks with binding energies, which are attributed to different types of containing nitrogen functional groups, including pyridinic-N (398.4 eV), pyrrolic-N (399.8 eV), graphitic-N 400.9 eV) and oxidized-N groups (402.4 eV) [50], and the results are listed in Table 2. Compared with ZC, the total nitrogen content of modified ZCs samples is increased. The percentage of nitrogen in ZC, ZCN200, ZCN400, and ZCN600 was calculated from XPS spectrum to be around 11.04, 14.90, 14.50, and 13.55. The nitrogen content declined from 14.90% to 13.55% when the heat treatment temperature rises from 200 °C to 600 °C, revealing that high temperature contributes to the decomposition of nitrogen-containing functional groups. The higher the temperature is, the less the amount of pyrrolic-N group is, even the ZC activated at 600 °C, the amount of the pyrrolic-N less than ZC. As the temperature of the heat treatment increase, the content of pyridinic-N group increases (see Table 2), indicating that pyrrolic-N is converted into pyridinic-N. The reason is that pyrrolic-N occurs for dehydrogenation to produce pyridinic-N at high temperature. 

### 3.2. Acetone Adsorption on Different Carbon Materials

**Figure 7:**
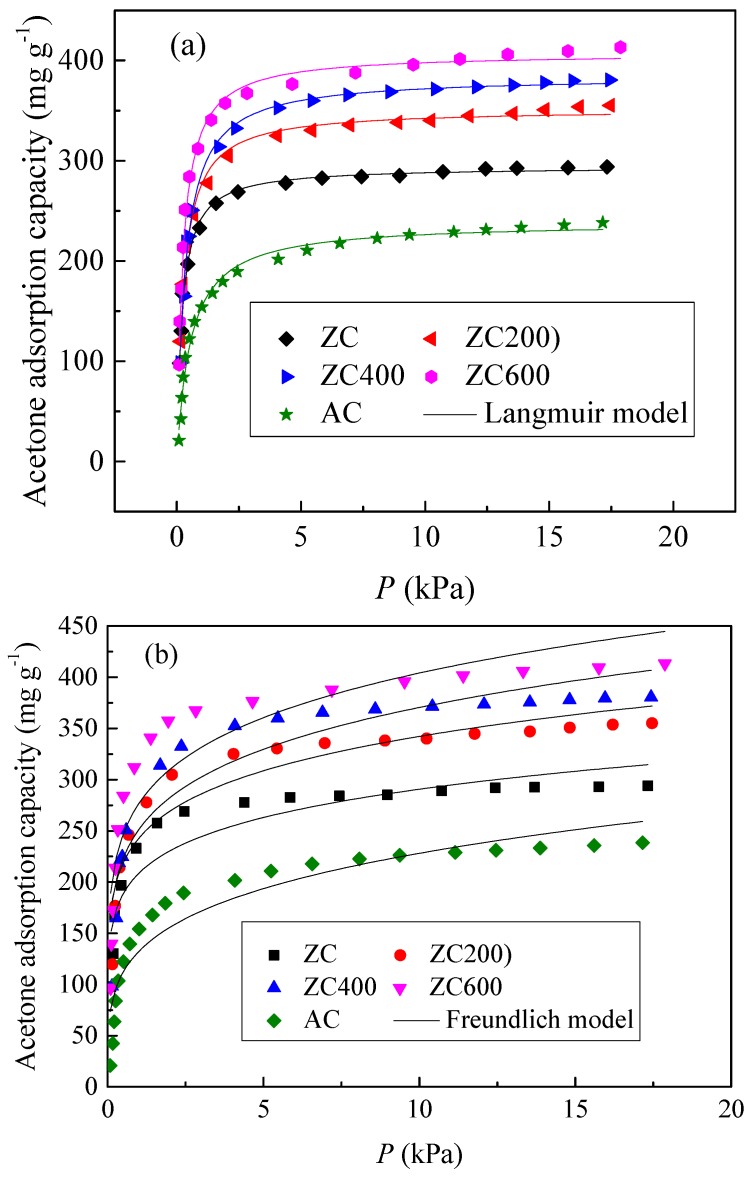

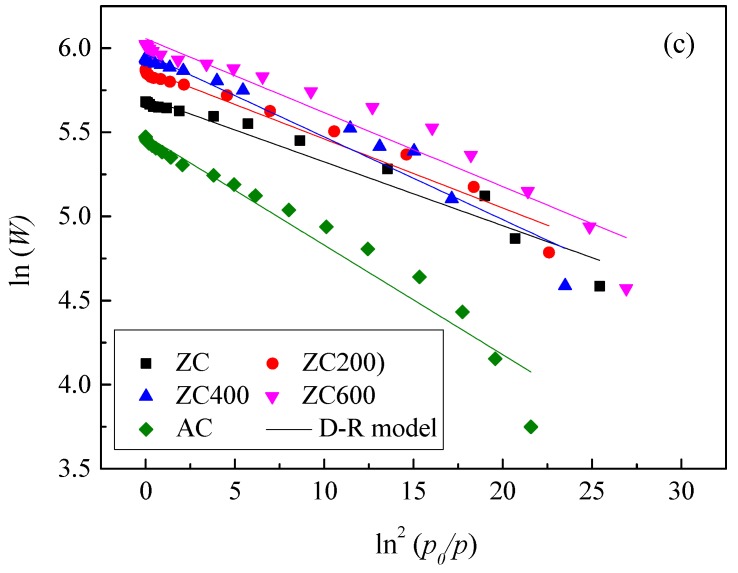
Adsorption isotherms were measured and then fitted with acetone on ZC, ZCNT and AC. (**a**) Langmuir model; (**b**) Freundlich model; (**c**) D–R model.

To quantitatively study the adsorption performance of acetone to activated carbon, it is necessary to use the appropriate isotherm model to further explain the data. Adsorption isotherms are the most important relationship between adsorbents and gas molecules. It also plays an important role in determining the maximum adsorption capacity. The isotherm of adsorbent with high concentration of acetone was obtained. The adsorption isothermal data were fitted with two-parameter models, Langmuir and Freundlich [51] which are expressed as:

(1) $$\mathrm{Langmuir},\;q=\frac{q_{\max}K_{L}p}{1+K_{L}p}$$

(2) $$\mathrm{Freundlich},\;q=K_{f}(p{)}^{1/n}$$

where *p* is the adsorption pressure of acetone, *q* is the adsorption capacity (mg g^−1^), *K_L_* *q*_max_ are Langmuir isotherm constants, where *q*_max_ signifies the theoretical monolayer capacity, *K_f_* (mg g^−1^) is the Freundlich constant and 1/*n* (dimensionless) is the heterogeneity factor. The adsorption isotherms obtained at 25 °C and predictions of the Langmuir and Freundlich models are shown in Figure 7. The corresponding characteristic parameters of the models along with the correlation coefficients are given in Table 3. From Table 3, determination coefficient, *R*^2^ of Langmuir equation is >0.99, indicating the good agreement with the experimental data for these adsorbents.

The Dubinin–Radushkevich (D–R) equation was used to describe experimental equilibrium adsorption characteristics of carbon materials and to interpret the adsorption behavior of acetone at 298 K. The equation representing the Dubinin–Radushkevich (D–R) model is expressed as [52]:

(3) $$W=W_{0}\exp\left[-{\left(\frac{RT\ln(p_{0}/p)}{E}\right)}^{2}\right]$$

where *W* is the adsorption capacity of the adsorbate, *W*_0_ is the maximun capacity available for adsorbate (mg g^−1^), *E* is the chacteristic adsorption energy (J mol^−1^), *R* is the ideal gas constant (8.314 J mol^−1^K^−1^), *T* is the absolute temperature (*K*), *p*_0_ is the saturated vapor pressure of the adsorbate at the adsorption temperature, and *p* is the vapor pressure of the test adsorbate. Equation (3) can be linearized and expressed as:

(4) $$\ln\;W=\;\ln\;W_{0}-\;{\left(\frac{RT}{E}\right)}^{2}\ln^{2}\left(\frac{p_{0}}{p}\right)$$

Figure 7c shows plots of ln (*W*) versus ln^2^(*p*_0_/*p*) for acetone adsorption on five carbon materials and applies linear regression to the experimental data, *W*_0_ and *E* can be calculated from the intercept on the ordinate and the slope of the linear-regression line, respectively. Compared to the Freundlich model, the D–R model can better represent the current data, but worse than the Langmuir model.

**Figure 8:**
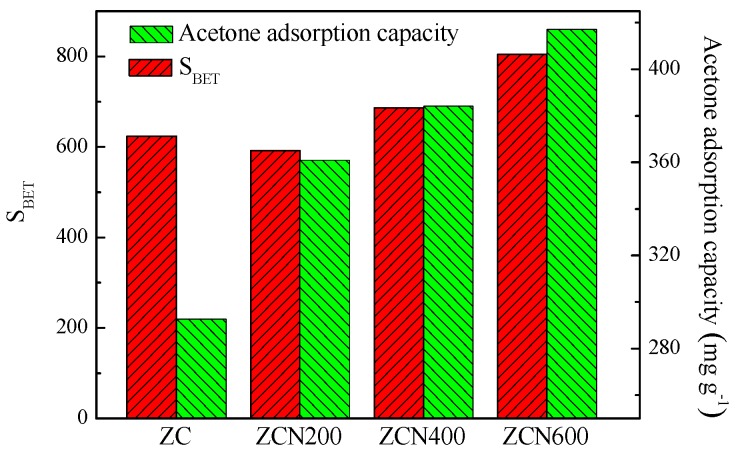
The relationship between BET specific surface area of the carbon and acetone saturated adsorption capacity.

It is generally believed that a higher acetone adsorption capacity depends on the large specific area of adsorbents. However, the adsorption of acetone on adsorbents in this work may yield a different result. The relationship between acetone saturated adsorption capacity and specific surface area is shown in Figure 8. It is clear that ZC having higher specific surface area than ZCN200 (see Table 1) provides the lower acetone saturated adsorption capacity (see Table 3). Compared with ZC, ZCN200 has higher acetone saturated adsorption capacity of 360.9 mg g^−1^, which increases by 17.4%, indicating that surface treatment by ammonia improves the affinity between acetone and carbon surfaces. Therefore, acetone saturated adsorption capacity of the ZCs samples is not closely related with the specific surface area. The reason may be that nitrogen groups in ZCN200 might increase polar character of the carbon surface and promote the affinity between acetone molecules and the carbon surface. Enhanced acetone adsorption of ZCN400 and ZCN600 is due to the improvement in nitrogen content and specific surface area.

**Figure 9:**
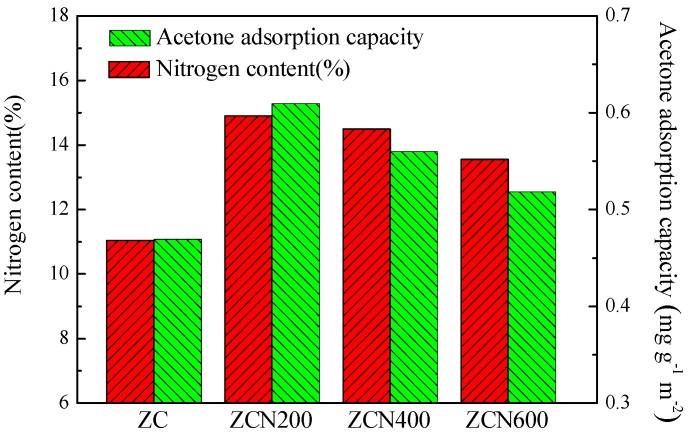
The relationship between nitrogen content of the carbon and the acetone saturated adsorption capacity based on unit surface area.

In order to investigate the effect of nitrogen functional groups on the adsorption of acetone, the adsorption capacity was normalized with respect to the surface area to exclude the effect of surface area of carbon materials. Figure 9 shows the relationship between the nitrogen functional groups and the acetone saturated adsorption capacity based on unit surface area of ZCs samples. In Figure 8, it can be clearly seen, acetone saturated adsorption capacity based on unit surface area of the carbon is associated with total nitrogen content of ZC, ZCN200, ZCN400, and ZCN600 samples. It can be inferred that acetone saturated adsorption capacity would be improved further for the ZCs samples associated with more nitrogen content, revealing the important role of the polar surface nitrogen-containing groups in acetone adsorption, which may be the anchor sites for acetone adsorption.

**Figure 10:**
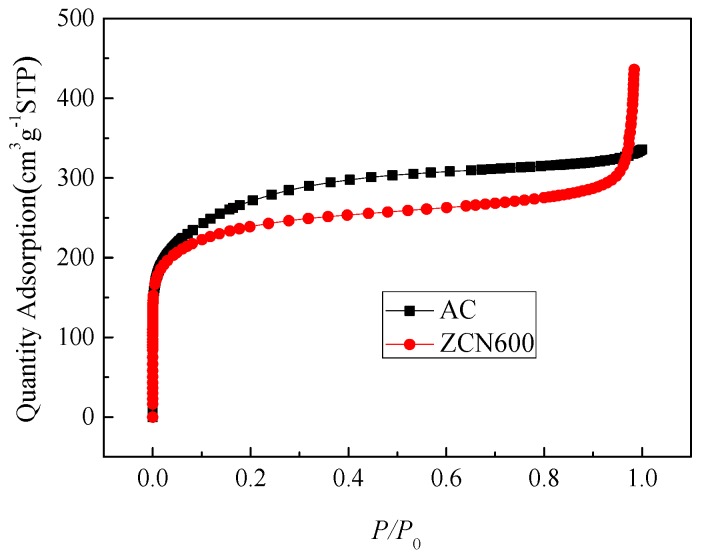
N_2_-sorption isotherms.

For comparison, commercially available AC was tested for acetone adsorption. From Figure 10, AC is essentially microporous, and its textural parameters are also listed in Table 1. Clearly, the acetone adsorption of ZCN600 is higher than that of AC (Table 3). Compared with textural parameters of AC, it is observed that the ZCN600 had higher acetone-adsorption capacity than that of AC, although AC has a much larger surface area and microporous volume (1.1 and 1.2 times larger than ZCN600). Compared with AC, the higher acetone-adsorption capacity of ZCN600 reveals that nitrogen-containing functional groups play an important role in acetone adsorption. After normalization of the acetone adsorption with surface area, it could be deduced that ZCN600 shows superior affinity to acetone than AC, leading to a much better acetone adsorption performance. The high adsorption is attributed to the affinity of polar interaction between acetone and polar nitrogen groups at room temperature. 

**Figure 11:**
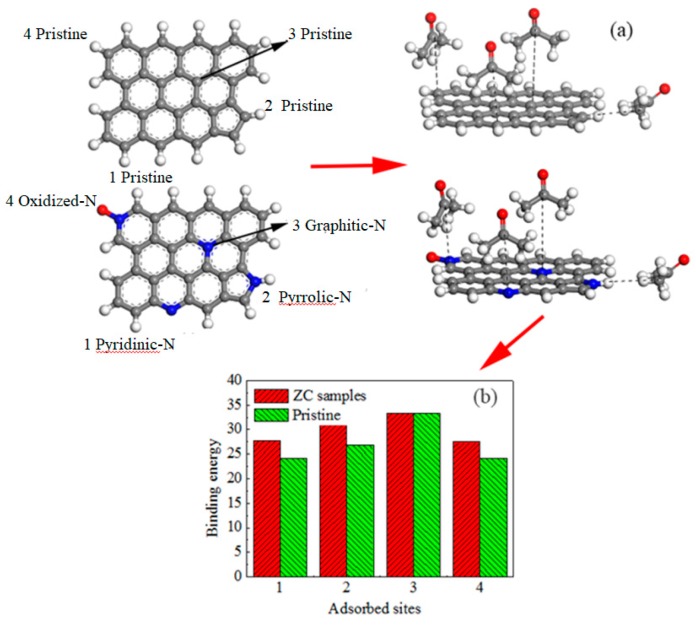
Theoretical models for (**a**) N-doped carbon surface and pure carbon surface (red ball: oxygen atom; blue ball: nitrogen atom; grey ball: carbon atom; small grey ball: hydrogen atom); (**b**) binding energies at different adsorption sites.

To understand the mechanism of acetone adsorption on the N-doped porous carbon, we use DMol^3^ for density functional theory (DFT) calculations. Figure 11 shows the geometric configurations for all the N-containing functional groups and total energy for all the N-containing functional groups. In these functional groups, polar interactions between acetone and N-containing functional groups are formed due to the donation of electronic charges with the hydrogen atom in acetone [37]. 

The computational results show that binding energies are different at different positions. The greater the binding energy Δ*E* (kJ mol^−1^) has a stronger adsorption affinity. As can be seen in Figure 10c, compared with pristine carbon, pyridinic-N, pyrrolic-N, and oxidized-N have higher bonding energies of 27.62, 30.65, and 27.45 kJ mol^−1^, which increased by 14.9%, 17.6%, and 14.1%, respectively, indicating the effects of N-containing functional groups on acetone adsorption heat on porous carbon. These results reflect that the N-containing functional groups, pyridinic-N, pyrrolic-N, and oxidized-N in particular, play an important role in acetone adsorption. Among them, pyrrolic-N is the best. From computational results above, it can be seen that the introduction of pyrrolic-N, pyridinic-N, and oxidized-N into the carbon surface efficiently promotes polar interactions between the N-containing functional groups and acetone molecular, which explains why the ZCN600 possesses a high acetone saturated adsorption capacity of up to 417.2 mg g^−1^. 

## 4. Conclusions

In summary, we have prepared ZIF-derived N-doped porous carbon (ZC) with micropore and macropore structure. In addition, the specific surface area and surface polarity of ZC was improved through ammonia treatment. ZCN-600 possesses a very high acetone saturated adsorption capacity of up to 417.2 mg g^−1^ at 25 °C. The acetone saturated adsorption capacity of the ZCs samples is not only closely related with the specific surface area and pore volume, but also with the N content of the carbons. Quantum chemical analysis reveals that the introduction of pyrrolic-N, pyridinic-N, and oxidized-N into a carbon surface facilitates the polar interaction between acetone molecules and the N-containing functional groups, which account for the high acetone adsorption of the porous carbon.

## Figures and Tables

**Table 1:** Porous structure parameters of the ZCs samples and AC.

Samples	*S*_BET_	*S*_mic_	*S*_mes_ + *S*_mar_	*V*_total_	*V*_mic_	*V*_mes_ + *V*_mar_	Yield
	m^2^ g^−1^	m^2^ g^−1^	m^2^ g^−1^	mL g^−1^	mL g^−1^	mL g^−1^	(%)
ZIF-8	1400	1370	30	0.71	0.65	0.06	-
ZC	624	453	171	0.46	0.21	0.25	31.7
ZCN200	592	422	170	0.43	0.20	0.23	31.2
ZCN400	686	514	172	0.53	0.24	0.29	30.3
ZCN600	805	627	178	0.62	0.29	0.33	26.5
AC	898	605	293	0.48	0.36	0.12	-

**Table 2:** Surface composition determined by XPS.

Sample	Concentration (at. %)						
	C	O	N	Pyridinic-N	Pyrrolic-N	Graphitic-N	Oxidized-N
ZC	85.5	3.46	11.46	4.75	2.16	3.04	1.06
ZCN-200	83.04	2.46	14.50	6.50	3.11	3.68	1.59
ZCN-400	81.92	3.18	14.90	6.84	2.65	3.59	1.42
ZCN-600	82.15	4.31	13.55	6.94	1.80	3.66	1.15

**Table 3:** Parameters of the Langmuir, Freundlich and D–R fitting results.

Samples	Langmuir		Freundlich				D–R		
	*q*_max_/mg g^−1^	*K*_L_/m^3^ g^−1^	*R*^2^	*Kf*/mg g^−1^	1/*n*	*R*^2^	*W*_0_/mg g^−1^	*E*/kJ mol^−1^	*R*^2^
ZC	292.7	2.59	0.9914	174.7	4.79	0.9135	300.0	12.5	0.9549
ZCN200	360.9	1.86	0.9964	203.3	4.56	0.8976	354.5	12.2	0.9636
ZCN400	384.2	1.59	0.9929	223.2	4.9	0.8743	389.3	11.2	0.9480
ZCN600	417.2	2.14	0.9933	239.9	4.46	0.8913	426.9	11.8	0.9321
AC	237.9	1.54	0.9937	127.1	4.10	0.9087	239.8	9.70	0.9538
